# Comparison of laboratory-based and non-laboratory-based WHO cardiovascular disease risk charts: a population-based study

**DOI:** 10.1186/s12967-022-03336-4

**Published:** 2022-03-16

**Authors:** Fatemeh Rezaei, Mozhgan Seif, Abdullah Gandomkar, Mohammad Reza Fattahi, Fatemeh Malekzadeh, Sadaf G. Sepanlou, Jafar Hasanzadeh

**Affiliations:** 1 Department of Social Medicine, Jahrom University of Medical Sciences, Jahrom, Iran; 2 Department of Epidemiology, School of Health, Shiraz University of Medical Sciences, Shiraz, Iran; 3 Non-Communicable Disease Research Center, Shiraz University of Medical Sciences, Shiraz, Iran; 4 Gastroenterohepatology Research Center, Shiraz University of Medical Sciences, Shiraz, Iran; 5 Digestive Disease Research Center, Digestive Disease Research Institute, Tehran University of Medical Sciences, Tehran, Iran; 6 Research Centre for Health Sciences, Institute of Health, School of Health, Department of Epidemiology, Shiraz University of Medical Sciences, Shiraz, Iran

**Keywords:** Risk assessment, Cardiovascular diseases, WHO risk chart, Agreement, Iran

## Abstract

**Background:**

Determining the risk of Cardiovascular Disease (CVD) is a necessity for timely preventive interventions in high-risk groups. However, laboratory testing may be impractical in countries with limited resources. This study aimed at comparison and assessment of the agreement between laboratory-based and non-laboratory-based WHO risk charts models.

**Methods:**

This study was performed using the baseline data of 8138 participants in the pars cohort study who had no history of CVD and stroke. The updated 2019 WHO model was used to determine the 10-year fatal and non-fatal CVD risks. In general, there are two types of new WHO risk prediction models for CVD. The scores were determined based on age, sex, smoking status, diabetes, Systolic Blood Pressure (SBP), and total cholesterol for the laboratory-based model and age, sex, smoking status, SBP, and Body Mass Index (BMI) for the non-laboratory-based model. The agreement of these two models was determined via kappa statistics for the classified risk (low: < 10%, moderate: 10–< 20%, high: ≥ 20%). Correlation coefficients (r) and scatter plots was used for correlation between scores.

**Results:**

The results revealed very strong correlation coefficients for all sex and age groups (r = 0.84 for males < 60 years old, 0.93 for males ≥ 60 years old, 0.85 for females < 60 years old, and 0.88 for females ≥ 60 years old). In the laboratory-based model, low, moderate, and high risks were 76.10%, 18.17%, and 5.73%, respectively. These measures were respectively obtained as 77.00%, 18.08%, and 4.92% in the non-laboratory-based model. Based on risk classification, the agreement was substantial for males < 60 years old and for both males and females aged ≥ 60 years (kappa values: 0.79 for males < 60 years old, 0.65 for males ≥ 60 years old, and 0.66 for females ≥ 60 years old) and moderate for females < 60 years old (kappa = 0.46).

**Conclusions:**

The non-laboratory-based risk prediction model, which is simple, inexpensive, and non-invasive, classifies individuals almost identically to the laboratory-based model. Therefore, in countries with limited resources, these two models can be used interchangeably.

## Background

Cardiovascular Diseases (CVDs) are the most important public health problems worldwide, with higher disproportionate consequences in developing countries. In 2015, 422.7 million cases with CVDs and 17.92 million CVD-related deaths occurred worldwide [1]. In Iran, nearly 50% of premature deaths have been attributed to CVDs [2]. Classification of people at risk of CVDs and its impact on the choice of preventive interventions is necessary for high-risk groups. Calculating the overall risk of CVDs to identify high-risk individuals can be cost-effective in Low- and Middle-Income Countries (LMICs) that have limited health resources [3, 4].

Up to now, several risk prediction models have been developed around the world to determine the overall risk of CVDs. These models aim to estimate the probability of a particular disease now or in the future. Determining the risk of CVDs has become essential in the prevention of these diseases and clinical trials [5]. One of the risk prediction models for CVDs is WHO risk charts. The first WHO risk charts were introduced in 2007. These charts estimate the 10-year risk of fatal and non-fatal CVDs for people without CVDs. They also classify each individual into different risk groups, so that they can manage their situations by modifying the lifestyle or medications, if needed [6]. In 2019, WHO updated the risk charts based on validated risk prediction models to estimate the CVDs risk in 21 Institute for Health Metrics and Evaluation (IHME) Global Burden of Disease (GBD) areas [7]. The new risk prediction models were calibrated using the data obtained from the GBD study including estimates for LMIC. The external validity of this model has been confirmed in several cohorts [8]. The previous risk charts published by the WHO provided CVDs risk for 14 global regions. There are two new WHO risk prediction models for CVDs: (1) a laboratory-based model that includes age, sex, smoking status, SBP), history of diabetes, and total cholesterol and (2) a non-laboratory-based model including age, sex, smoking status, SBP, and BMI [7]. Choosing the non-laboratory-based model depends on the setting and goals of CVDs risk assessment, which may be the best model for deciding on primary and secondary preventive interventions in the general community. In many LMICs, laboratory measurements may not be available at primary healthcare centers or people may not afford the tests due to their high costs. Therefore, the non-laboratory-based model can be used [9].

In Iran, some studies have used different risk prediction models. In Iran, the American College of Cardiology/American Heart Association (ACC/AHA) and World Health Organization/International Society of Hypertension (WHO/ISH) risk models have been used for risk prediction [10, 11]. In another study, the agreement between laboratory-based and non-laboratory-based models was calculated using the Framingham risk score for the pars cohort population [12]. However, no study has compered the laboratory-based and non-laboratory-based WHO CVD risk prediction charts.

It is important to find out whether the laboratory- and non-laboratory-based risk models provide similar estimates of the 10-year CVDs risk in an individual. Thus, the present study aims at comparison and evaluation of the agreement between the updated 2019 WHO CVD risk based on laboratory-based and non-laboratory-based risk models in a large population.

## Methods

### Study population

This cross-sectional study was conducted using the baseline data of the pars cohort study, which was proceeded in Valashahr and its neighboring villages in southern Iran in fall 2012. Valashahr includes about 40.000 residents. Details of the pars cohort study have already been published [13]. Briefly, it was conducted on 9264 individuals aged 40–75 years from 2012 to 2014. The participants’ demographic characteristics including age, sex, lifestyle variables including smoking, and disease history including heart disease, stroke, hypertension, diabetes were collected using structured questionnaires by trained interviewers. The physical exam was done by trained physicians and nurses who were employed at the Pars Cohort Center. The physical examination included anthropometric indexes (height and weight) and the determination of systolic and diastolic blood pressure, as well. Staffs were native residents. They were familiar with residents. So, people were able to communicate with them confidently and effectively for more information about any issue. Totally, 8138 cases without a history of CVDs or stroke were recruited.

### CVDs risk

In the present study, the 10-year risk of CVDs was calculated using WHO laboratory-based and non-laboratory-based models. The laboratory-based model included age, sex (male/female), SBP (mmHg), smoking status (current/other), history of diabetes (yes/no), and cholesterol (mmol/l), while the non-laboratory-based model included age, sex, SBP (mmHg), smoking status (current/other), and BMI.

Smoking status was determined through the interviews. In addition, the history of diabetes was determined by the previous history of the disease or Fasting Blood Sugar (FBS) ≥ 126 mg/dL. BP was measured for each participant using a mercury sphygmomanometer after a 5-min rest. BP was measured twice, with a 10-min interval, from each arm and the mean BP was recorded. Cholesterol was tested in the laboratory. Finally, BMI was computed by dividing weight by height squared (kg/m^2^).

### Statistical analysis

Percentage was reported for the categorical variables and mean and Standard Deviation (SD) for quantitative ones. Chi-square and t-test were used for categorical and continuous variables, respectively.

The agreement between the laboratory-based and non-laboratory-based models was determined using two models based on the type of risk score, which could be continuous or categorical. Correlation coefficients and scatter plots of the predicted individual-level risk of fatal and non-fatal CVDs were used to present the correlation between the laboratory-based and non-laboratory-based CVDs risk scores. Accordingly, the correlation coefficients 0.00–0.19, 0.20–0.39, 0.40–0.59, 0.60–0.79, and 0.80–1.0 indicated  a very weak correlation between the two variables, weak correlation, moderate correlation, strong correlation, and very strong correlation, respectively [14]. The correlation coefficients and scatter plots were presented based on gender and age groups (< 60 and ≥ 60 years).

In the WHO risk model, the predicted risk is classified into five groups; i.e., very low (< 5%), low (5% to < 10%), moderate (10% to < 20%), high (20– < 30%), and very high (≥ 30%). In this study, it was classified into three groups: low (< 10%), moderate (10% to < 20%), and high (≥ 20%). The agreement between the classified risk of laboratory-based and non-laboratory-based models was evaluated using kappa statistics. The agreement less than odds was indicated by kappa values < 0, slight agreement by kappa values between 0.01 and 0.20, fair agreement by kappa values between 0.21 and 0.40, moderate agreement by kappa values 0.41–0.60, substantial agreement by kappa values 0.61–0.80, and almost complete agreement by kappa values 0.81–0.99 [15]. Statistical analyses were performed with Statistical Package for Social Science (IBM SPSS Statistics for Windows, Version 23.0. Armonk, NY: IBM Corp) and Stata Statistical Software (Stata 14 for windows, Stata Corp., College Station, TX, USA). P-values less than 0.05 were considered as statistically significant.

## Results

In this study, 4349 participants (53.44%) were female and the mean age of the participants was 51.65 ± 9.06 years. In addition, the prevalence of smoking was higher among males compared to females. The prevalence of hypertension and diabetes were higher in females than in males. The mean diastolic blood pressure was slightly higher in males than in females. However, the means of SBP, High Density Lipoprotein (HDL), Low Density lipoprotein (LDL), cholesterol, and BMI were higher in females compared to males.

**Fig. 1:**
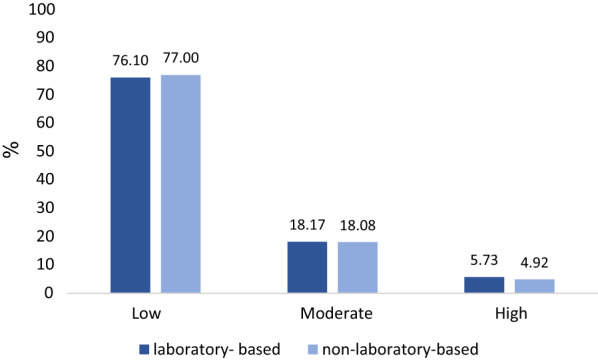
The percentage of cardiovascular risks classified according to laboratory-based and non-laboratory-based models

The mean 10-year CVDs risk in the total population was slightly higher in the laboratory-based model than in the non-laboratory-based model (7.60 ± 6.41 vs. 7.49 ± 5.98). In both models, the mean 10-year CVDs risk was higher in males compared to females (Table 1). The risk classification of laboratory-based and non-laboratory-based models was very similar (Fig. 1). Based on the results, 5.73% and 4.92% were high risk in the laboratory-based and non-laboratory-based models, respectively.

**Table 1:** The participants’ characteristics

Variables	Males (n = 3789)	Females (n = 4349)	Total (n = 8138)
	N (%)	N (%)	N (%)
Age range (years)			
< 60	3023 (79.78)	3434 (78.96)	6457 (79.34)
≥ 60	766 (20.22)	915 (21.04)	1681 (20.66)
Smoking (now)			
No	2644 (69.78)	4321 (99.36)	6965 (85.59)
Yes	1145 (30.22)	28 (0.64)	1173 (14.41)
Hypertension			
No	3517 (92.82)	3580 (82.32)	7097 (87.21)
Yes	272 (7.18)	769 (17.68)	1041 (12.79)
Diabetes			
No	3585 (94.62)	3871 (89.01)	7456 (91.62)
Yes	204 (5.38)	478 (10.99)	682 (8.38)
DBP (mean mmHg ± SD)	73.44 ± 11.61	72.92 ± 11.91	73.16 ± 11.77
SBP (mean mmHg ± SD)	110.78 ± 17.50	111.54 ± 19.64	111.19 ± 18.68
HDL (mean mmol/l ± SD)	1.40 ± 0.30	1.56 ± 0.34	1.49 ± 0.33
LDL (mean mmol/l ± SD)	2.65 ± 0.82	2.86 ± 0.89	2.78 ± 0.86
Chol (mean mmol/l ± SD)	4.85 ± 1	5.24 ± 1.08	5.06 ± 1.06
TG (mean mmol/l ± SD)	1.74 ± 1.30	1.74 ± 1.10	1.74 ± 1.20
BMI (kg/m^2^)	24.31 ± 4.08	27.05 ± 4.73	25.77 ± 4.64
Laboratory-based CVDs risk score (10- year, %) (mean ± SD)	8.31 ± 7.06	6.98 ± 5.71	7.60 ± 6.41
Non-laboratory-based CVDs risk score (10-year, %) (mean ± SD)	8.15 ± 6.68	6.92 ± 5.23	7.49 ± 5.98

*DBP* diastolic blood pressure, *SBP* systolic blood pressure, *HDL* high density lipoprotein, *Chol* cholesterol, *TG* triglyceride, *BMI* body mass index

### Correlation coefficient

The correlation coefficients between the laboratory-based and non-laboratory-based WHO CVDs risks have been presented in Table 2. The results revealed a very strong positive correlation between the laboratory-based and non-laboratory-based models amongst males (r = 0.94, p < 0.001) and females (r = 0.94, p < 0.001). A very strong positive correlation coefficient was also observed among both males and females in the two age groups. Accordingly, a very strong positive correlations were found in males < 60 years old (r = 0.84, p < 0.001), males ≥ 60 years old (r = 0.93, p < 0.001), females < 60 years old (r = 0.85, p < 0.001), and females ≥ 60 years old (r = 0.88, p < 0.001).

**Table 2:** Pearson correlation of the predicted individual-level risk of fatal and non-fatal cardiovascular disease using the non-laboratory-based with laboratory-based model

	N	Correlation coefficient (r)	P-value	Comment*
Males				
All males	3789	0.94	< 0.001	Very strong positive
< 60 years old	3023	0.84	< 0.001	Very strong positive
≥ 60 years old	766	0.93	< 0.001	Very strong positive
Females				
All females	4349	0.94	< 0.001	Very strong positive
< 60 years old	3434	0.85	< 0.001	Very strong positive
≥60 years old	915	0.88	< 0.001	Very strong positive

^*^ 0.00–0.19 “very weak,” 0.20–0.39 “weak,” 0.40–0.59 “moderate,” 0.60–0.79 “strong,” 0.80–1.0 “very strong”

**Fig. 2:**
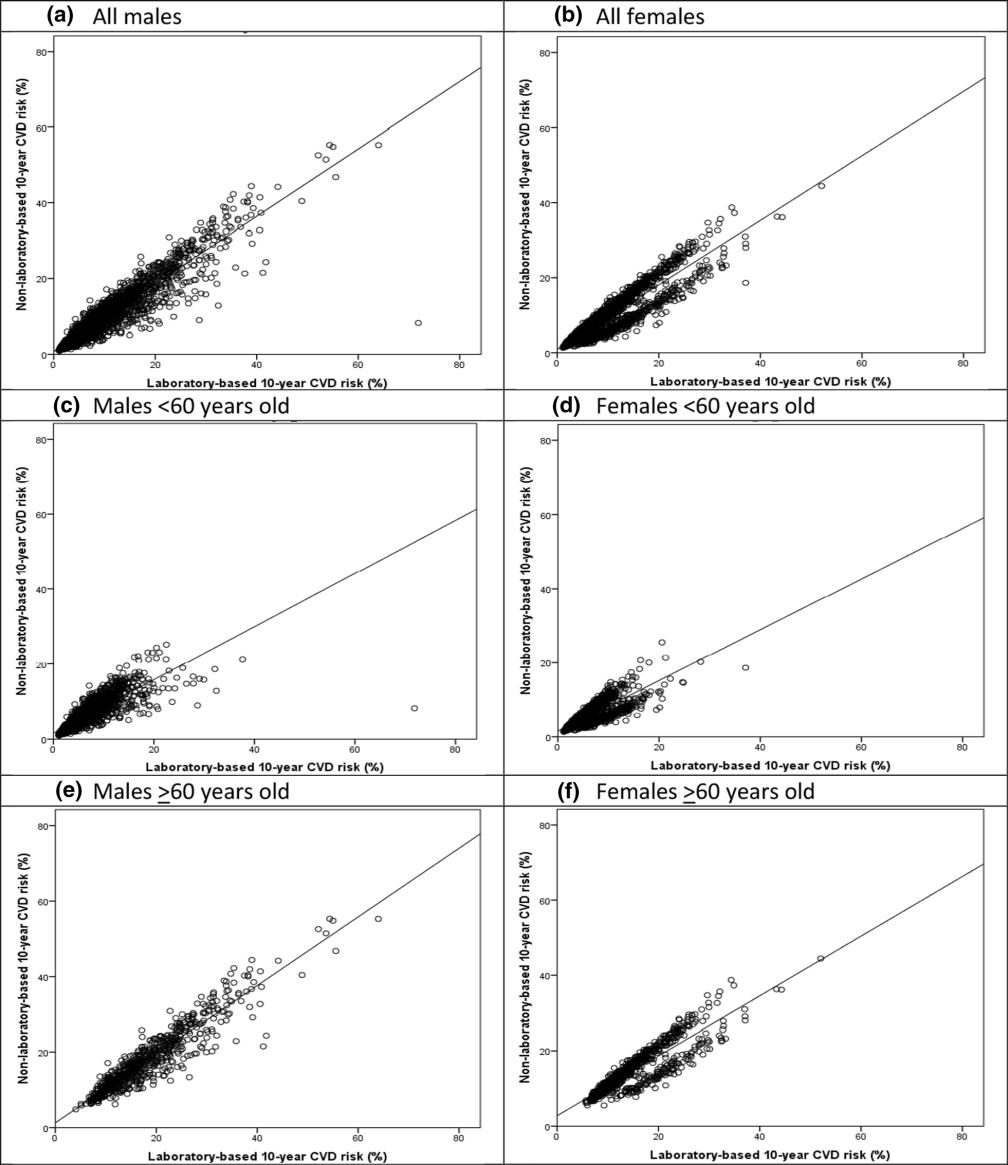
Scatter plot of the predicted individual-level risk of fatal and non-fatal cardiovascular disease using the non-laboratory-based versus laboratory-based model. **a** All males, **b** All females, **c** Males < 60 years old, **d** Females < 60 years old, **e** Males ≥ 60 years old, **f** Females ≥ 60 years old

Scatter plots of the predicted individual-level risk of fatal and non-fatal CVDs using the laboratory-based and non-laboratory-based scores for males and females in the two age groups have been shown in Fig. 2. Accordingly, a very strong positive correlation was found amongst males and females in the two age groups.

### Categorical agreement

In the total population, the agreement between the two risk scores according to the risk score categories was 91.37% (kappa = 0.77, Standard Error (SE) = 0.00). Categorical agreements between the two risk scores have been presented in Table 3 (males) and Table 4 (females). For males, the agreement between the risk categories was 91.39% (kappa = 0.79, SE = 0.01). In addition, the number of males in the high-risk group was higher in the laboratory-based model than in the non-laboratory-based model (272 vs. 245). Moreover, the agreement was 92.95% for the males aged < 60 years (kappa = 0.65, SE = 0.02) and 85.25% for those aged ≥ 60 years (kappa = 0.75, SE = 0.02). The agreement was substantial for the two age groups. Considering females, the agreement between the risk categories was 91.35% (kappa = 0.75, SE = 0.01). Besides, the number of females in the high-risk group was higher in the laboratory-based model compared to the non-laboratory-based model (194 vs. 155). Furthermore, the agreement was 94.20% for the females < 60 years old (kappa = 0.46, SE = 0.03) and 80.65% for those ≥ 60 years old (kappa = 0.66, SE = 0.02). The agreement was moderate and substantial for the females aged < 60 and ≥ 60 years, respectively.

**Table 3:** Agreement between the laboratory-based and non-laboratory-based risk scores according to the grouped risk in males

Laboratory-based risk category	Non-laboratory-based risk category				Agreement (%)	Kappa (SE)
	Low	Moderate	High	Total		
All males						
Low	2671	116	0	2787	91.39	0.79 (0.01)
Moderate	113	582	35	730		
High	5	57	210	272		
Total	2789	755	245	3789		
< 60 years old						
Low	2583	92	0	2675	92.95	0.65 (0.02)
Moderate	94	221	6	321		
High	5	16	6	27		
Total	2682	329	12	3023		
≥ 60 years old						
Low	88	24	0	112	85.25	0.75 (0.02)
Moderate	19	361	29	409		
High	0	41	204	245		
Total	107	426	233	766

**Table 4:** Agreement between the laboratory-based and non-laboratory-based risk scores according to the grouped risk in females

Laboratory-based risk category	Non-laboratory-based risk category				Agreement (%)	Kappa (SE)
	Low	Moderate	High	Total		
All females						
Low	3299	107	0	3406	91.35	0.75 (0.01)
Moderate	178	545	26	749		
High	1	64	129	194		
Total	3478	716	155	4349		
< 60 years old						
Low	3146	47	0	3193	94.20	0.46 (0.03)
Moderate	141	86	2	229		
High	1	8	3	12		
Total	3288	141	5	3434		
≥ 60 years old						
Low	153	60	0	213	80.65	0.66 (0.02)
Moderate	37	459	24	520		
High	0	56	126	182		
Total	190	575	150	915		

## Discussion

In this study, the new WHO model updated in 2109 was used to predict the 10-year risk of CVDs. These models help CVDs risk prediction in primary health centers and can result in public health interventions. In many LMICs, laboratory testing is not always available in primary care centers due to the cost and lack of resources. Hence, a non-laboratory model is used to determine the risk of CVDs.

In the present study, the correlation between the laboratory-based and non-laboratory-based models was measured using correlation coefficients. In addition, kappa statistics were employed to determine whether the non-laboratory-based model could replace the laboratory-based one. Several studies have recommended the use of WHO risk charts in low-income countries [3, 16]. In some studies, the agreement between different CVDs risk prediction models has been investigated [17, 18]. Moreover, some other studies have evaluated the agreement or correlation between laboratory-based and non-laboratory-based Framingham risk scores [12, 19, 20].

According to the correlation coefficients and scatter plots in the present study, there was a strong correlation between the two risk scores in both males and females. The correlation coefficients were also strong for all sex and age groups (r = 0.84 for the males < 60 years old, 0.93 for the males ≥ 60 years old, 0.85 for the females < 60 years of age, and 0.88 for the females ≥ 60 years old). However, the correlation coefficients were higher in elderly people. In the same line, another study indicated a high correlation between the mean scores of laboratory-based and non-laboratory-based models [20]. Another study also revealed a strong positive correlation between lipid-based and BMI-based-models in predicting the Framingham risk score [21].

Due to the fact that the risk was classified into three groups of low (< 10%), moderate (10% to < 20%), and high (≥ 20%) in the present study, the agreement between the classified risks of laboratory-based and non-laboratory-based models was evaluated using kappa statistics. According to the results, the agreement was better in males than in females. Among males, the agreement was substantial in both age groups. On the other hand, the agreement was moderate in the females < 60 years old and substantial in those ≥ 60 years of age. In another study, the agreement between the two risk scores of Framingham laboratory-based and non-laboratory-based models was 74.8% and the kappa statistic was 0.63, which was slightly lower than the overall agreement in the current research [22]. Another study performed in Sri Lanka revealed a good agreement between the cholesterol-based and BMI-based WHO/ISH models (kappa = 0.804) [23].

The comparability of the laboratory-based and non-laboratory based CVDs risk estimation has been shown in settings where resources are limited, which can further optimize cost-effective strategies [20]. For instance, the Heart Wellness Study indicated that the BMI-based risk score could be used to distinguish low-risk people and potentially decrease additional laboratory testing [24]. Other studies also emphasized that the non-laboratory-based model could accurately predict the consequences of CVDs [25, 26].

The results of the present study showed that there were a larger number of people in the high-risk group in the laboratory-based model than in the non-laboratory-based model (5.73% vs. 4.92%). In contrast, another study found that there were a larger number of people in the high-risk group in the Framingham BMI-based risk model than in the cholesterol-based model. In that study, the agreement was good for all sex and age groups, except for older men that was fair [19]. Another study reported that the BMI-based model overestimated the risk of CVDs [27]. In the present study, however, a large proportion of the high-risk participants in the laboratory-based model were also identified in the non-laboratory-based model. In another study, laboratory-based and non-laboratory-based models indicated that 9.4% and 12.7% of the participants were high-risk. In addition, a larger number of people were in the high-risk group in the non-laboratory-based model compared to the laboratory-based model [20]. On the contrary, the findings of the research carried out in Sri Lanka demonstrated that more participants belonged to the high-risk group in the non-laboratory-based model compared to the laboratory-based model (10.7% vs. 9.5%) [23]. The techniques used for measurement of BMI, BP, and cholesterol can result in the underestimation or overestimation of the CVDs risk, eventually increasing or decreasing the risk scores of these models [19]. However, cohort studies in Iran are conducted by skilled staff and accurate tools that can be reliable. Furthermore, ethnicity, socioeconomic status, and genetics in different geographical areas can increase or decrease the risk of CVDs. Another reason for the discrepancy among the results is that most studies comparing the agreement between laboratory-based and non-laboratory-based models have used the Framingham risk prediction model, while the WHO model was utilized in the current study. The Framingham risk model is based on a single Caucasian cohort.

The present study findings demonstrated that the non-laboratory-based model could be used instead of the laboratory-based model. Thus, CVDs risk assessment can be done using the non-laboratory-based model in settings with low resources [28]. Kariuki et al. also disclosed that the non-lab based model decreased the costs by 11% compared to the laboratory-based model [29]. Therefore, the non-laboratory-based model is recommended, especially in settings with limited resources where extensive laboratories are not available and it is not economically feasible to perform laboratory tests.

## Study strengths and limitations

The main strength of the present study was the large sample size and the use of carefully collected data from a population-based study. Therefore, the findings can be generalized. To the best of our knowledge, this was the first study comparing two laboratory-based and non-laboratory-based models using the updated 2019 WHO risk chart in a large population. However, this cross-sectional study is based on the baseline data of a cohort study. Thus, a longitudinal study with a 10-year follow-up period is suggested to be conducted to validate laboratory-based and non-laboratory-based risk models. Another limitation is the lack of HbA1c test for diabetes. Because, in low-income countries, the HbA1c test is expensive, especially for studies that are performed on a large population.

## Conclusions

The present study results revealed that the correlation coefficients were very strong for all sex and age groups. A substantial agreement was also observed between the scores when classified as low, moderate, and high. Thus, the non-laboratory-based risk prediction model classified individuals almost similarly to the laboratory-based model. This model, which is measured without a blood test, can lead to the beginning of the treatment without the need for additional costs or the inconvenience of laboratory testing. Additionally, the healthcare providers who have limited resources and time in primary health centers can use this model to evaluate the risk inexpensively and to make treatment decisions in a timely manner. Therefore, in countries with limited resources where individuals cannot afford laboratory tests, these two models can be used interchangeably. Yet, future longitudinal cohort studies with 10-year follow-up periods are required to validate laboratory-based and non-laboratory-based risk models for the pars cohort population.

## Data Availability

The data used in this study are available from the corresponding author on reasonable request.

## Abbreviations

CVD: Cardiovascular disease
WHO: World health organization
LMICs: Low- and Middle-Income Countries
IHME: Institute for health metrics and evaluation
GBD: Global burden of disease
ACC/AHA: American College of Cardiology/American Heart Association;
ISH: International Society of Hypertension
BP: Blood pressure
DBP: Diastolic blood pressure
SBP: Systolic blood pressure
FBS: Fasting blood sugar
HDL: High density lipoprotein
Chol: Cholesterol
TG: Triglyceride
BMI: Body mass index
